# Underwater soundscape in Seaview Bay, Antarctica, and triple ascending trill of the leopard seal (*Hydrurga leptonyx*) underwater vocalizations

**DOI:** 10.1002/ece3.70038

**Published:** 2024-07-25

**Authors:** Dong‐Gyun Han, Jee Woong Choi, Jong‐U Kim, Jeong‐Hoon Kim, Hyoung Sul La

**Affiliations:** ^1^ Research Center for Ocean Security Engineering and Technology Hanyang University ERICA Ansan Republic of Korea; ^2^ Division of Ocean & Atmosphere Sciences Korea Polar Research Institute Incheon Republic of Korea; ^3^ Oceansounds Incorporation Ansan Republic of Korea; ^4^ Department of Marine Science and Convergence Engineering Hanyang University ERICA Ansan Republic of Korea; ^5^ Department of Military Information Engineering Hanyang University ERICA Ansan Republic of Korea; ^6^ Division of Life Sciences Korea Polar Research Institute Incheon Republic of Korea; ^7^ Department of Polar Science University of Science and Technology Daejeon Republic of Korea

**Keywords:** acoustic characteristics, leopard seal vocalization, passive acoustic monitoring, Seaview Bay, triple ascending trill, underwater soundscape

## Abstract

The underwater soundscape was recorded in Seaview Bay off Inexpressible Island, Ross Sea region Marine protected area, for 3 days in December 2021. Leopard seal *Hydrurga leptonyx* vocalizations were a prominent sound source that led to variations in ambient sound pressure levels in a frequency range of approximately 150–4500 Hz. Among the 14 call types previously identified, except ultrasound vocalizations, six types of broadcast calls were classified, and their acoustic characteristics were analyzed. We focused on the acoustic characteristics of four low‐frequency calls, clustered in a relatively narrow bandwidth, which have been relatively less studied. We identified a new call type of a triple ascending trill consisting of three trill parts, expanding upon the findings of previous studies. The audio data extracted from leopard seal vocalization videos, recorded by a monitoring camera on sea ice, enhanced the reliability of identifications of the underwater triple ascending trill. We present the unique results of underwater passive acoustic monitoring conducted at Seaview Bay, designated as Antarctic Specially Protected Area No 178. Our results could contribute to the development of detection and localization algorithms for leopard seal vocalizations and can be used as fundamental data for studies related to the vocalization and behavior of this species.

## Graphical abstract

Identify a new call of the leopard seal, the triple ascending trill, in the RSR MPA.

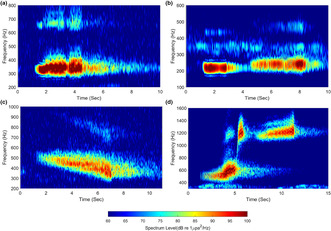

## 1 INTRODUCTION

Many marine mammal species have been observed in the Ross Sea region marine protected area (Ainley, 2010; Erbe et al., 2017; Giorli & Pinkerton, 2019), which is the most pristine region in the world and one of the least human‐impacted marine environments. This region, known for its biodiversity hotspots, includes the world's largest marine protected area established by the Commission for the Conservation of Marine Living Resources CCAMLR (2016). Marine mammals, the high trophic level of the food web, are a group representing the health of the marine ecosystem (Nelms et al., 2019; Ross, 2000), and their abundance and diversity could be important factors determining the state of the ocean as sentinels. Because marine mammals spend most of their time underwater, the traditional visual detection method is complemented by underwater acoustic monitoring, which can record long‐term data independent of the time of day and weather conditions. Passive acoustic monitoring (PAM) is an effective method for investigating the presence, distribution, and behavior of soniferous marine mammals, especially in polar environments where the observation conditions are poor.

The leopard seal *Hydrurga leptonyx* is classified as “least concern” on the International Union for Conservation of Nature (IUCN) Red List of threatened species (Hückstädt, 2015). This species, rarely sighted and usually observed close to pack ice, is a top predator in the ecosystem of the Ross Sea and is a highly vociferous during the austral spring and summer (Rogers et al., 1996; Stirling & Siniff, 1979). Ray first reported one underwater vocalization of leopard seals in 1970. Stirling and Siniff quantitively described four call types in 1979 within a frequency range of 150 and 5900 Hz, and vocalizations have been categorized into two groups: “broadcast calls” and “local calls” (Rogers et al., 1996). “Broadcast calls” made by mature species and not intended for a particular receiver may function for mating and/or territorial indication at a long distance and consist of high double trill (HDT), medium single trill (MST), low double trill (LDT), descending trill (DT), hoot with single trill (HST), and hoot (H). Highly stereotyped broadcast calls are observed only from December to January when female seals exhibit elevated estradiol levels associated with sexual receptivity (Rogers et al., 1996). Call types of medium double trill (MDT) and ascending trill (AT) have also been reported (Klinck, 2008; Stirling & Siniff, 1979), and call repertoires and acoustic characteristics show geographic differences (Klinck, 2008; Kreiss et al., 2013; Rogers et al., 1995). “Local calls,” which are relatively less studied, are associated with close interactions between two seals and consist of growl, snort, thump pulse, noseblast, roar, and blast (Rogers et al., 1996). Ultrasonic sounds up to 164 kHz were also recorded from captive seals as they chased fish (Thomas et al., 1983). Adult male seals produce highly stereotyped broadcast calls, and subadult males have more variant calls (Rogers, 2007; Rogers, 2017). In addition, the source level assuming spherical spreading was estimated to be from 153 to 177 dB re 1 μPa (Rogers, 2014).

Most studies on the acoustic characteristics of leopard seal vocalizations have predominantly concentrated on broadcast calls rather than local calls, with a particular focus on HDT and LDT, which constitute the largest portion of the underwater vocal repertoire (Kreiss et al., 2013; Rogers & Cato, 2002; Van Opzeeland et al., 2010). They have primarily utilized spectrograms to estimate acoustic characteristics such as frequency bandwidth (encompassing maximum and minimum frequencies), peak frequency, call duration, and pulse repetition rate (PRR). The inherent variability in both the amplitude and structure of vocal signals, coupled with the spatiotemporal variability of background noise levels, may pose challenges in estimating these characteristics and in the effective detection of call signals. Therefore, it is necessary to apply adaptive extraction methods that consider the composition and variation of the vocal signals to achieve more precise acoustic characteristics. In terms of detection methods, automatic detection methods for marine mammal vocalizations in long‐term PAM data have been suggested (Erbe & King, 2008; Miller et al., 2021), but their application to leopard seals has been limited (Klinck, 2008; Klinck et al., 2008). Recent efforts in developing algorithms using artificial intelligence have been actively conducted (Shamir et al., 2014; Shiu et al., 2020), and well‐labeled datasets based on manual detection results are imperative for facilitating the development of robust models. Therefore, acoustic data observed underwater, which are more inaccessible than in terrestrial environments, are valuable resources prior to the development of automated detection and analysis algorithms, especially for protected species such as leopard seals.

Our study presents the first results of underwater passive acoustic monitoring on Seaview Bay off Inexpressible Island, designated the Antarctic Specially Protected Area (ASPA) No. 178 by the Secretariat of the Antarctic Treaty (ATS) (Ministry of Environment (MOE), 2020). We focused on leopard seal vocalizations, which are rarely studied but commonly recorded in this region during the mating season, and the acoustic characteristics and temporal variations of each call type were investigated. An unmanned aerial vehicle (UAV) and a monitoring camera, which have been used actively for marine environment monitoring recently, were operated together to overcome the limitations of underwater acoustic monitoring, which requires acoustic source classification based on previous reports.

## 2 METHODS

### 2.1 Data collection

**FIGURE 1:**
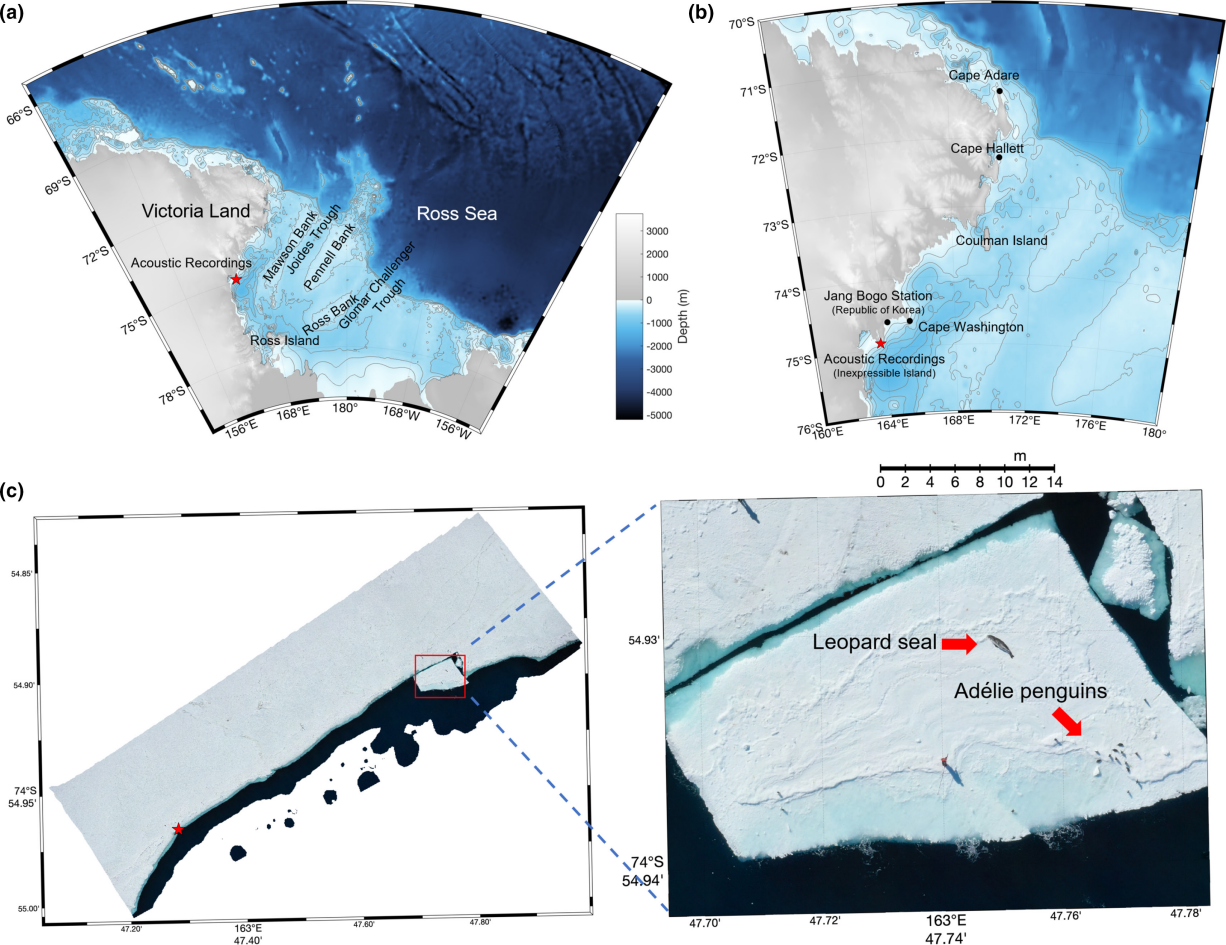
(a) Map of the Ross Sea region and Victoria Land, (b) enlarged view of the coastal area, and (c) UAV image of the acoustic measurement area taken on December 9, showing the sea ice edge (red asterisk indicates the deployment location of the acoustic recorder). A single leopard seal observed on the pack ice is shown in an expanded image of the red square box.

Passive acoustic monitoring was conducted on the east coast of Inexpressible Island, Ross Sea, Antarctica (Figure 1a,b), from 15:00 on December 9 to 21:00 on December 11, 2021 (54 h), using local time (UTC + 13). An autonomous passive acoustic recorder (μAURAL, Multi‐Electronique Inc.) was deployed on the sea ice edge (74° 54.966'S, 163° 47.284'E) at a depth of 30 m, which is approximately 1.5 km from the coastline (red asterisk in Figure 1c). The acoustic recorder was scheduled to record continuously in 10‐min WAV files with 24‐bit resolution and a 96,000 Hz sampling rate. Excluding data during deployment and recovery, the 324 WAV files had a time interval of 2–3 s between each file, resulting in a total missing data period of 14‐min. The receiving voltage sensitivity of the hydrophone is −165 dB re1V/μPa and almost flat with a ±2 dB error in the frequency range of 10 Hz to 10 kHz (De Robertis & Wilson, 2011). Therefore, the acoustic data were analyzed within the given frequency band, with frequencies above 10 kHz used only as a reference. Figure 1c shows aerial photographs taken by a UAV (Mavic 2 Pro, DJI) depicting the measurement area and the location of an autonomous passive acoustic recorder (red asterisk), including the spatial distribution of the wildlife and sea ice. The length of the sea ice edge in the photo was approximately 1.8 km, and a single leopard seal and Adélie penguins, *Pygoscelis adeliae*, were observed on the pack ice in the red square box of the expanded scale of the photo. The female seal was over 2 m long (Figure S1). The vocalizations of this individual were not included in the underwater acoustic data recorded at the time, and its contribution to the entire acoustic data cannot be confirmed. A monitoring camera (A6600, SONY) with a 20–200 mm lens was used to take videos and photos.

### 2.2 Underwater soundscape analysis

All data processing, analysis, and visualization were conducted using MATLAB 2021a (Mathworks, Inc.). The underwater soundscape was analyzed in the time‐averaged spectrum levels and spectrograms. The spectrum levels were estimated by the power spectral density (PSD) in units of dB re 1 μPa^2^/Hz (Han et al., 2021), which was computed from 1 min of acoustic data with a 1‐s Hanning window, 50% overlap and a fast Fourier transform length of 96,000 points. The spectral probability density, which represents percentile spectra (Merchant et al., 2013), was then calculated to evaluate temporal variations in the sound pressure levels and the impact of leopard seal vocalizations on the underwater soundscape in the measurement region. The spectrogram was represented by stacking the intensity‐averaged PSD in 10‐min intervals, as that depicted by higher temporal resolution was ineffective to show acoustic events over time.

### 2.3 Detection and classification of leopard seal vocalizations

During the analysis of the underwater soundscape, we confirmed that leopard seal vocalizations were prominent and persistent throughout the recording period, as evidenced by spectrograms from randomly selected WAV files. Consequently, we focused on detecting and classifying these vocalizations. All acoustic data files, recorded in 10‐min durations, were subjected to the detection process on both a waveform and a spectrogram in 1‐min intervals. Call signals, which are stronger than the background noise level and whose features could be clearly distinguished in the spectrogram, were manually detected, and then classified into HDT, MST, LDT, HST, DT, and AT based on the call types in previous studies (Klinck, 2008; Rogers, 2007; Stirling & Siniff, 1979). The call patterns of the five types were mostly consistent with those reported in previous studies except for AT, and the acoustic characteristics of each call type were extracted by a process that considered the detailed features of the signals. For low‐frequency vocalizations, uncertainty was accounted for by re‐detecting calls within 1‐h segments, counting the number of missed and false detections, and adjusting these values relative to the total recording times. Similarly, for HDT and MST, uncertainty was estimated by randomly extracting detected signals, counting the number of false detections, and adjusting these values relative to the corresponding time period.

### 2.4 Acoustic characteristics analysis

**FIGURE 2:**
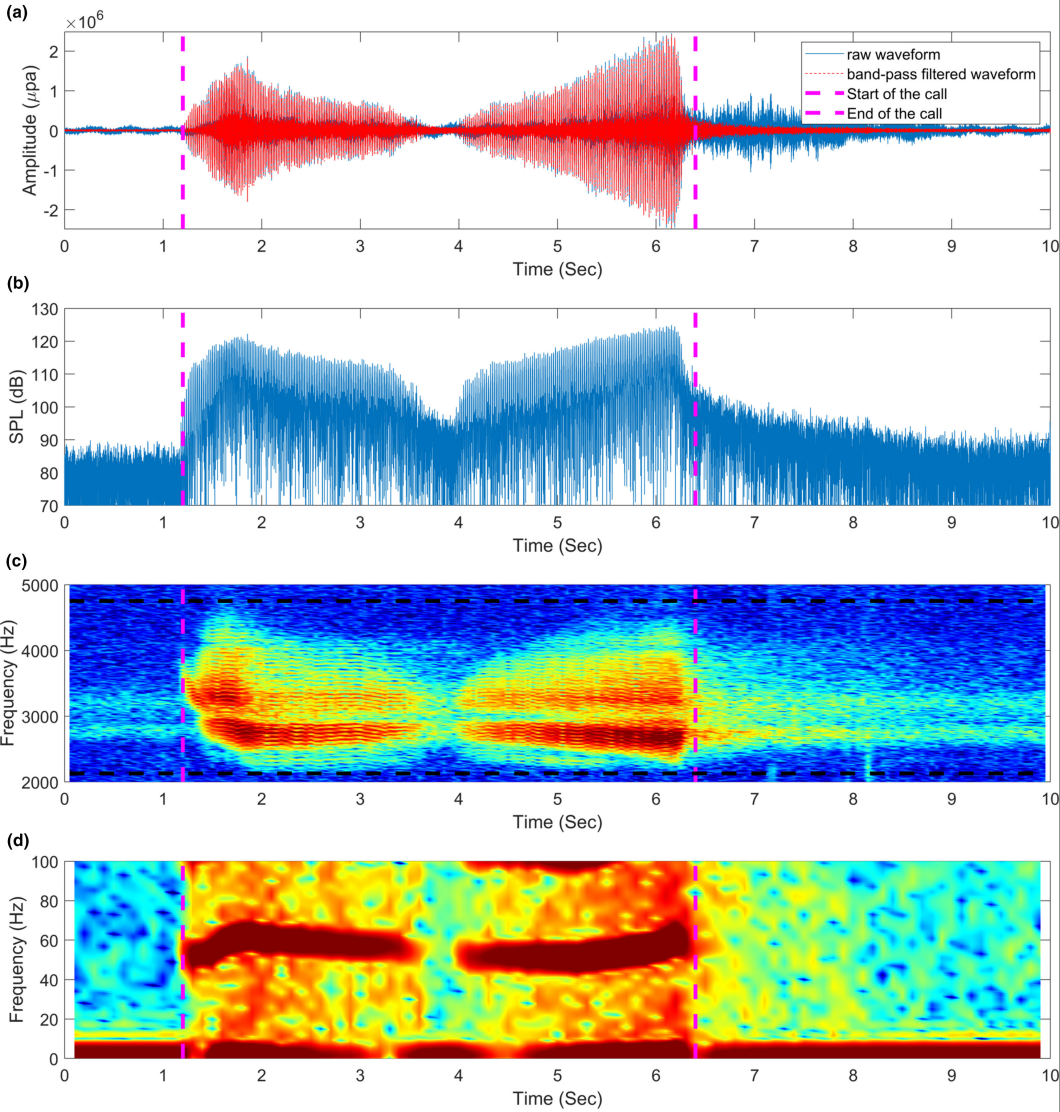
Analysis results of the high double trill as a function of time. The magenta dashed lines represent the beginning and ending points of the call, and the black dashed lines represent the maximum and minimum frequencies. (a) Waveforms (cyan solid: Raw waveform, red dashed: Bandpass‐filtered waveform), (b) sound pressure level, (c) spectrogram (48,000 fast Fourier transform points and 4800‐point Hanning window), and (d) spectrogram of the envelope waveform representing the amplitude modulation frequency (96,000 fast Fourier transform points and 19,200‐point Hanning window).

Acoustic characteristics of call duration, peak frequency, and maximum/minimum frequency were estimated from at least 100 sample signals of a single call. Because classified call datasets contained signals that either overlapped a couple of vocalizations or exhibited a low signal‐to‐noise ratio, we extracted acoustic characteristics from randomly selected sample signals containing a single call with a sufficient signal‐to‐noise ratio. The extraction of acoustic characteristics commenced by plotting figures of the waveform, spectrogram, sound pressure level, and spectrogram of the envelope waveform as a function of time (Figure 2). The call types of leopard seal analyzed in this study included trill sounds, which exhibited the amplitude modulation patterns. The amplitude modulation rate of the trill waveform is characterized by the pulse repetition rate, which is more effectively represented using the envelope‐spectrogram technique (Klinck et al., 2008).

The call duration was calculated from the difference between the start and end points of the call signal, which varied depending on the call type. For HDT, the start of the first trill and the end of the subsequent trill were aligned well with the beginning and ending points of amplitude modulation, respectively. Therefore, the initiation and termination of the call were determined by the start and end points of the amplitude modulation within the frequency range of 30–80 Hz (Figure 2d). For the MST, LDT, HST, DT, and AT, the end point of the call was designated the termination of the amplitude modulation pattern. However, determining the start of the amplitude modulation pattern was ambiguous because the amplitude modulation rate and energy of the trill at the beginning of the call either increased gradually or the amplitude modulation pattern did not appear when the call started with a narrowband component, like a hoot rather than a trill. Therefore, for these five call types, call initiation was determined when vocal characteristics were discernible and exceeded the background noise level in both the sound pressure level and the spectrogram. H is characterized by the absence of a trill, the start point of the call was manually determined at the point where the energy significantly increased in the sound pressure level, spectrogram, and PRR. The end point of the call was defined as the moment when strong energy ceased at an amplitude modulation frequency below 10 Hz. The hoot is discussed in Section 4.

In previous studies, the frequency bandwidth of calls was defined as ±20 dB around the peak frequency (Rogers et al., 1995), or specialized programs such as Raven Pro (Cornell lab) and Osprey (Mellinger & Bradbury, 2007) were utilized (Heimrich et al., 2021; Shabangu & Rogers, 2021). While we adhered to the previously established criterion of a ±20 dB bandwidth, this criterion was insufficient for HDT, MST and AT. For these three calls, the start and end frequencies that exceeded the background noise spectrum levels before and after the call signal were calculated as maximum and minimum frequencies, respectively. The peak frequency was determined as the frequency corresponding to the highest power value at the spectrum level, and this criterion was applied to every signal. More detailed features not mentioned in previous studies of each call are explained in Section 3.2.

## 3 RESULTS

### 3.1 Temporal variation in the underwater soundscape

**FIGURE 3:**
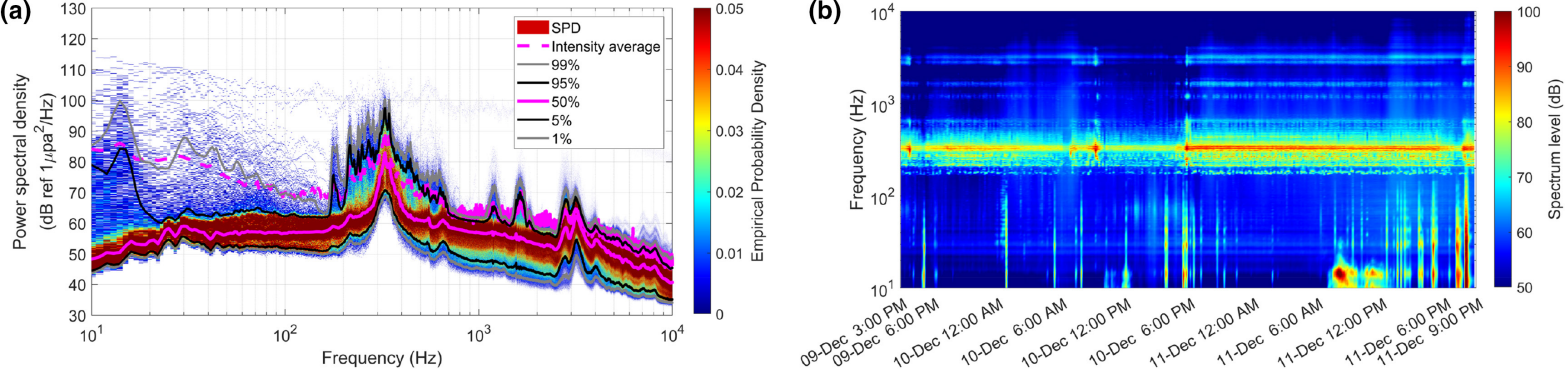
(a) The spectral probability density of the acoustic data measured over 54 h. The thick magenta lines represent the average values of the mean intensity spectrum level (dashed) and the median spectrum level corresponding to the 50th percentile (solid). (b) Spectrogram estimated from 10‐min intensity average spectrum levels.

The temporal variation in the underwater soundscape is shown by the spectral probability density and spectrogram in Figure 3. At the median spectrum level represented by the magenta solid line in Figure 3a, spectral peaks near 330 and 3200 Hz were conspicuous, and they seemed to be attributed to the calls of leopard seals. The sound pressure level of the most prominent peak at 328 Hz was 25 dB higher than the median spectrum level below 1 kHz, except in the bandwidth between 200 and 500 Hz, and this sound source was the dominant contributor to the increased ambient sound pressure level in this region. Relatively weak spectral peaks near 1200 and 1700 Hz were observed at the mean intensity spectrum level, represented by the magenta dashed line, but they were not sustained enough to be reflected at the median spectrum level. The feature represented by these four spectral peaks is also shown on the spectrogram in Figure 3b. These four spectral peaks exhibited almost synchronized occurrence patterns, with differences in sound pressure levels. They showed more prominent persistence on December 11 than on December 10 and presented relatively lower power from 10:00 to 18:00 on December 10. The temporal variations in the sound pressure levels are discussed in Section 3.3 using correlation with call counts of leopard seal vocalizations.

### 3.2 Call types of underwater vocalizations and their acoustic characteristics

**FIGURE 4:**
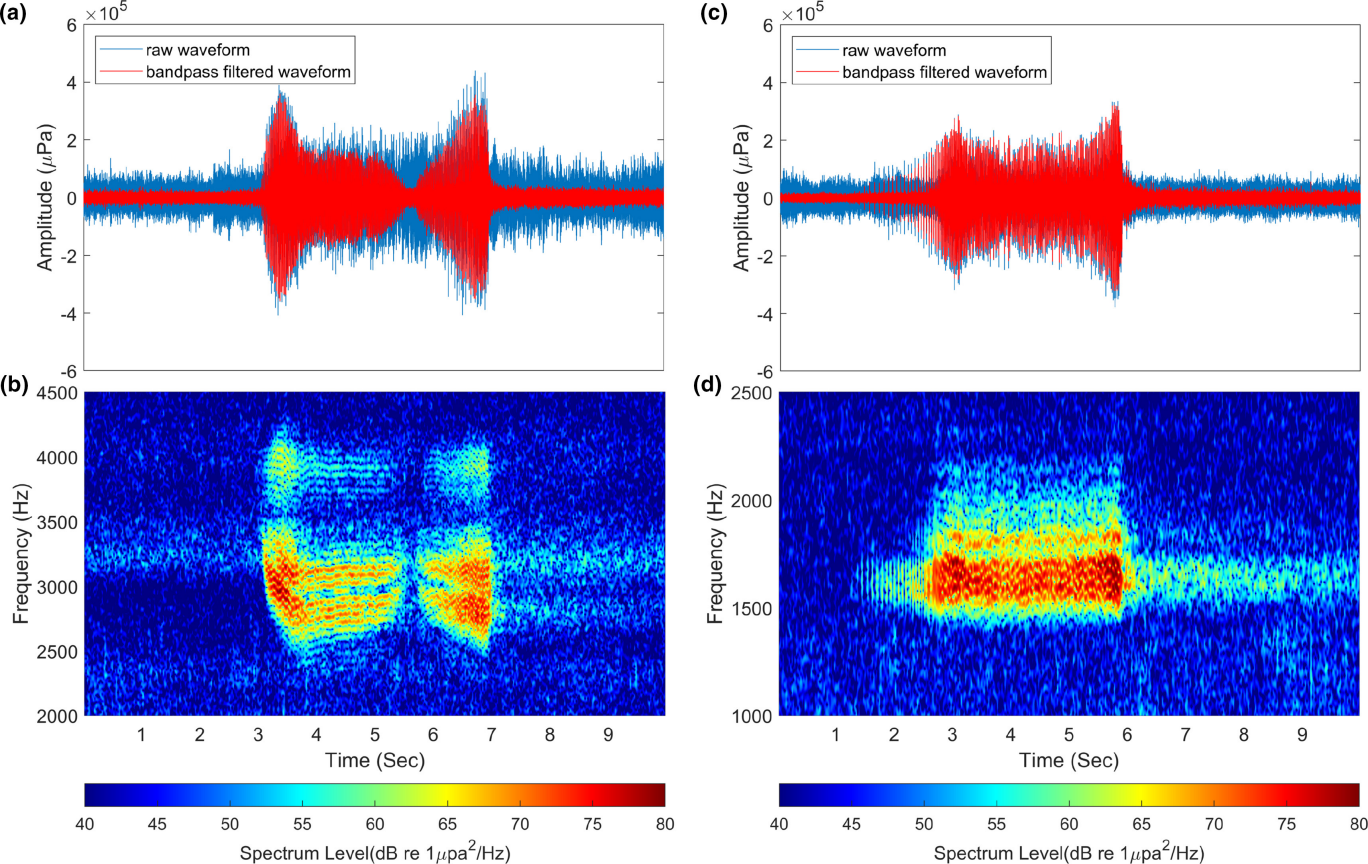
Typical waveforms and spectrograms of leopard seal vocalizations. (a, b) High double trill, (c, d) medium single trill. The raw waveform and the bandpass‐filtered waveform are shown by cyan and red solid lines, respectively (48,000 fast Fourier transform points and 4800‐point Hanning window).

**FIGURE 5:**
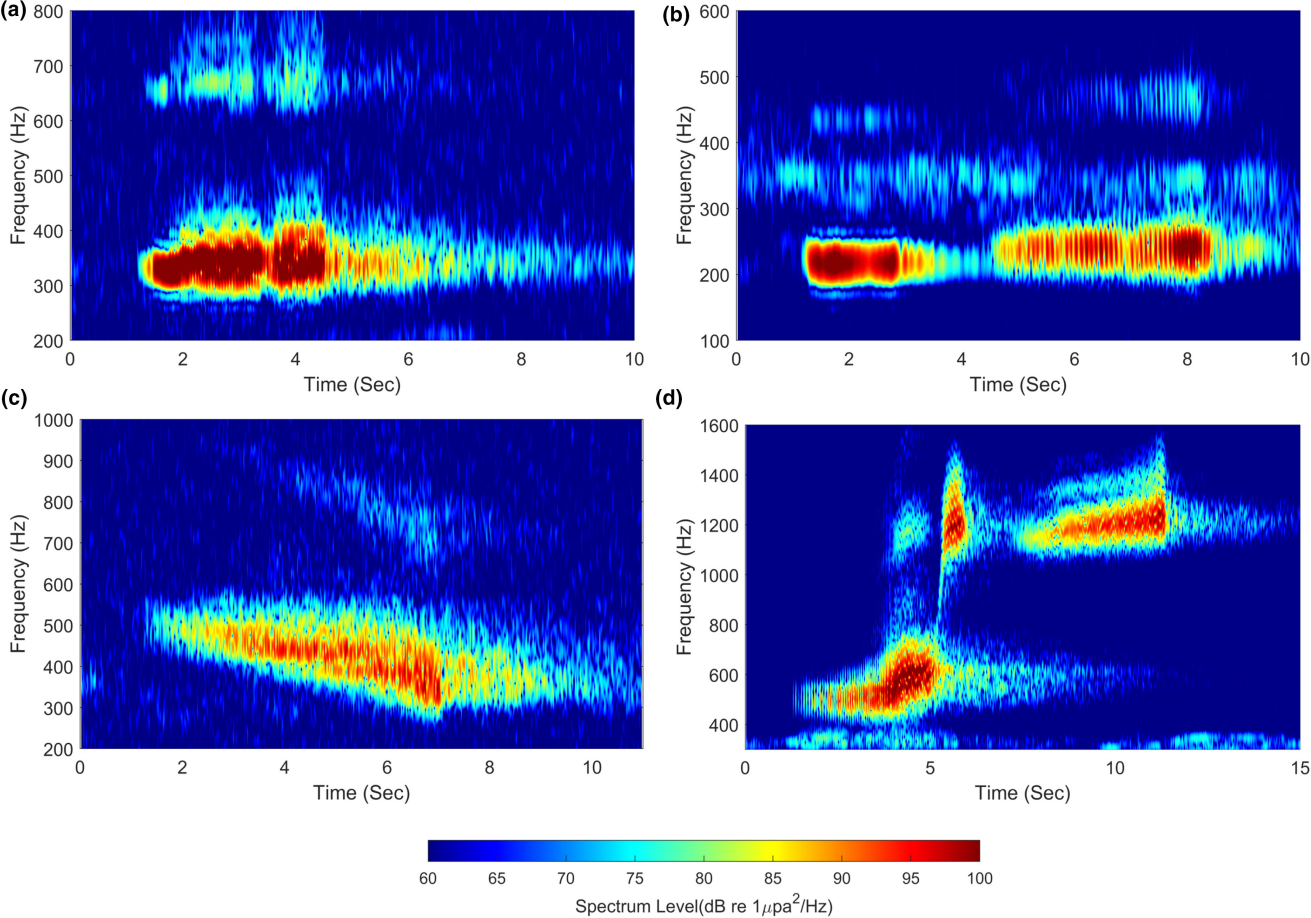
Spectrograms of low‐frequency calls of leopard seal vocalizations. (a) Low double trill, (b) hoot with single trill, (c) descending trill, and (d) triple ascending trill (48,000 fast Fourier transform points and 4800‐point Hanning window).

HDT and MST were observed in frequency bands between 2300 and 4500 Hz, and between 1300 Hz and 2400 Hz, respectively, and they were relatively stereotyped and well time‐resolved. Several vocalizations were clustered in relatively narrow bandwidths of approximately 100 and 500 Hz, and we detected and identified four call types: LDT, HST, DT, and AT. Over 54 h, 12,006 calls were detected across six types, and representative spectrograms of these six call types are shown in Figures 4 and 5. Low‐frequency vocalizations were the most frequent at 58%, followed by HDT (32%) and MST (10%). Among the low‐frequency vocalizations, the combined LDT (35%) and HST (13%) accounted for the greatest proportion at 48%, followed by DT (7%), and AT (3%). As reported in previous studies, HDT consisted of two trills in series, and MST was a single trill characterized by beginning with a progressively accelerating pulse repetition, thus causing a gradual increase in amplitude modulation frequency (Kreiss et al., 2013; Rogers, 2007; Rogers et al., 1996).

LDT, consisting of two trills in series, begins with a relatively short and narrowband component. The spectrum level of this initial narrow component is higher than that of the subsequent double‐trill parts, and trill parts exhibit a broader bandwidth of several 10s of Hz compared to that of the narrowband component. HST consisted of a relatively stronger single narrowband component followed by a single trill, which had a slightly higher center frequency than the narrowband component, with distinct time intervals separating them. DT, characterized by a center frequency that gradually decreases within approximately 290 and 570 Hz, shows a tendency for the amplitude modulation to slightly increase around 20 Hz. AT, previously reported as a single trill, was observed as three trills in series (hereafter referred to as triple ascending trill) in our measurements. A total of 362 triple ascending trills were recorded, each composed of three ascending trill parts in the frequency range of approximately 400–1540 Hz (Figure 5d). Single ascending trills were also observed sporadically with a significantly low signal‐to‐noise ratio, but they were not addressed in this study due to high uncertainty in detection. These issues are discussed in Section 4. The first ascending trill part, starting at approximately 400 Hz, was characterized by a progressively accelerating pulse repetition rate within the range of 10–60 Hz, similar to MST (Figure S2). The second ascending trill part showed a rapid increase in center frequency, connecting the other two trill parts within a relatively short duration. The last ascending trill part, with a center frequency slowly increasing within the frequency range of 1000–1500 Hz, exhibited a slow increase in the pulse repetition rate at approximately 40 and 50 Hz and its harmonic components. The acoustic characteristics of the six call types extracted from sample calls are listed in Table 1. The reverberation effects and amplitude modulation patterns of the calls are discussed in Section 4.

**TABLE 1:** Acoustic characteristics of leopard seal vocalizations extracted from sample calls and call counts detected during acoustic measurements.

	Peak frequency (Hz)	Minimum frequency (Hz)	Maximum frequency (Hz)	Duration (seconds)	Total call counts
High double trill (*n* = 110)	2822 ± 96	2372 ± 88	4121 ± 354	4.2 ± 1.2	3903 ± 8
Medium single trill (*n* = 113)	1641 ± 26	1379 ± 32	2305 ± 91	4.3 ± 0.3	1141 ± 2
Low double trill (*n* = 102)	329 ± 9	292 ± 7	389 ± 14	3.7 ± 0.6	4226 ± 401
Hoot with single trill (*n* = 100)	233 ± 18	209 ± 17	280 ± 19	6.3 ± 1.4	1516 ± 223
Descending trill (*n* = 104)	408 ± 27	299 ± 8	557 ± 13	5.4 ± 0.4	858 ± 211
Triple ascending trill (*n* = 111)	587 ± 37	420 ± 19^a^	1456 ± 85^b^	9.9 ± 0.6	362

*Note*: *n*, the number of call samples, acoustic characteristics: mean ± SD, call counts ± uncerntainty.

^a^ Component measured in the first ascending trill part.

^b^ Component measured in the third ascending trill part.

### 3.3 Temporal variation in call rate and its contribution to the soundscape

**FIGURE 6:**
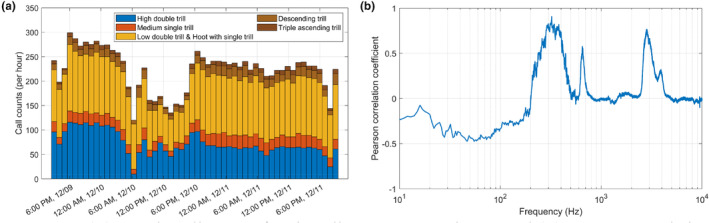
(a) Hourly call counts for six call types over 54 h, and (b) Pearson correlation coefficients between total call counts and spectrum levels as a function of frequency.

The call counts per hour, as presented in Figure 6a, were relatively lower from 05:00 to 17:00 on December 10. The Pearson correlation coefficient between the hourly call counts and sound pressure levels over 54 h showed a weak positive correlation (*r* = .40, *p* = .0029). Figure 6b represents the results of the correlation analysis between the hourly call counts and spectrum levels at 1‐Hz intervals. High correlations were observed at 323, 652, and 2809 Hz frequencies within specific bandwidths. The peak at 323 Hz, which closely resembles the 328 Hz peak in the median spectrum level of the underwater soundscape, exhibited a strong positive correlation with a coefficient of 0.9.

## 4 DISCUSSION

Our research was motivated by the objective of implementing passive acoustic monitoring in a region that is known for its well‐preserved marine environment. During the analysis, we identified leopard seal vocalization as the dominant sound source. Unlike many previous studies that have focused on vocalizations of target species from identified individuals from an ecological perspective, our research aimed to interpret the vocalizations produced by unidentified individuals of both sexes and ages after the measurements.

**FIGURE 7:**
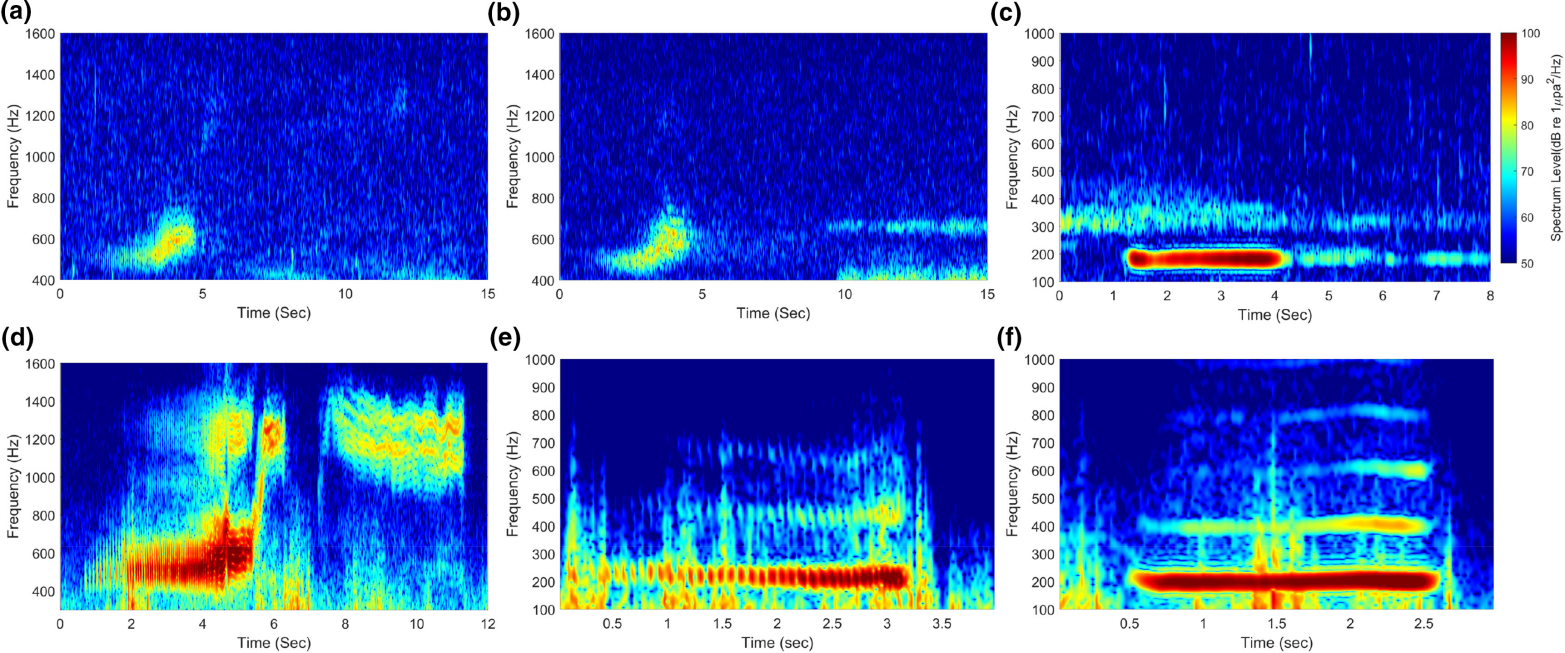
Spectrograms of leopard seal vocalizations recorded in (a–c) underwater and in (d–f) the air. (a) triple ascending trill, (b) ascending trill, (c) hoot, (d) triple ascending trill, (e) low single trill, and (f) hoot (underwater data: 48,000 fast Fourier transform points and 4800‐point Hanning window, airborne data: 48,000 fast Fourier transform points and 2400‐point Hanning window).

One of the most significant findings in our results was the identification of a new call type, the triple ascending trill. The ascending trill has been mentioned in only four previous studies (Klinck, 2008; Shabangu & Rogers, 2021; Thomas et al., 1995; Van Opzeeland et al., 2010). Specifically, Van Opzeeland et al. (2010) and Klinck et al. (2008) reported it as a single ascending trill with a spectrogram, while Thomas et al. (1995) described it as call 4, consisting of two or three components. Most of the ascending trills observed in our data comprised three distinguishable trill parts. In contrast, the single ascending trills recorded in our measurements had a very low signal‐to‐noise ratio, and some triple ascending trills exhibited faint second and third ascending trill parts (Figure 7a,b), making clear identification challenging. This suggests that AT is fundamentally composed of three parts, but weak single ascending trills might be recorded when the distance between the recorder and the individual is large. Consequently, the single ascending trill was excluded from detection and acoustic characterization due to its significantly higher detection uncertainty. To verify whether the triple ascending trill is a call of the leopard seal, we checked video data containing the airborne vocalizations of the male leopard seal, taken on December 12 after the underwater acoustic measurements for 26.9 min using a monitoring camera (48,000 Hz sampling rate). We extracted 55 vocal signals, which were divided into 7 call types—HDT, MST, LDT, DT, triple ascending trill (Figure 7d), low single trill (Figure 7e) and, hoot (Figure 7f)—from the 4.4‐min video, excluding indistinct signals due to the wind noise and sections without vocalizations. The call pattern of the triple ascending trill observed in the air was similar to that recorded underwater. The lack of the colorbar on the vocal spectrogram recorded in the air was due to our inability to obtain the receiving voltage sensitivity of the camera microphone from the manufacturer, which rendered quantitative analysis of the airborne acoustic data impossible. The airborne vocalizations exhibit more apparent trill patterns and harmonic components than those recorded underwater, with much less reverberation. Reverberation in the underwater acoustic waveguide lasts relatively longer compared to air due to interaction with ocean boundaries such as the sea surface and seafloor (Katsnelson et al., 2012). This supports the validity of the method for determining the end point of the call by the end point of the amplitude modulation pattern. While most of the HDT, MST, and LDT waveforms exhibited relatively distinct amplitude modulation patterns, those of the trill parts in HST, DT, and AT were not clearly visible. This difference may be caused by their vocal mechanisms, but further verification is required to confirm the reason. Since we focused on the underwater vocalizations of leopard seals and airborne data were used to support the observations of triple ascending trill, detailed information regarding airborne vocalizations can be found in previous studies (Rogers et al., 1995).

In our study, call types were categorized based on previous research; however, there were low single trill and LDT cases without a strong narrow component and indistinct double trill cases, similar to low single trill in the frequency band overlapping with LDT. As these were identified as variant calls of LDT in a previous study (Rogers, 2007), we did not classify them as separate call types. Additionally, HST exhibited large variations in the duration and interval of hoot and trill. Consequently, the uncertainty in the call counts of low‐frequency vocalizations, including DT, was high. Furthermore, single hoot was detected in all the acoustic data; however, due to the significant variability in its sound pressure level and the difficulty in distinguishing them from HST, they were not included in the call count, similar to the single ascending trill. From 101 sample signals of single hoot with relatively high signal‐to‐noise ratios, the estimated peaks, minimum frequencies, maximum frequencies, and call duration were 182 (±10 SD), 163 (±8 SD), 201 (±10 SD) Hz and 2.7 (±0.4 SD) seconds, respectively. A representative spectrogram of a single hoot is shown in Figure 7c. We also addressed that the process of calculating the upper and lower limit frequencies of the HDT, MST, and triple ascending trill, which are relatively broadband calls, based on their contrast against the ambient noise level, which is also a meaningful point of this study. Despite these efforts, the call rates and acoustic characteristics of each call type were estimated, acknowledging that the manual process of detecting calls and determining the start points of sample calls may be subject to uncertainty. In particular, call detection under low signal‐to‐noise ratio conditions remains technically challenging. We have established call datasets, which is clustered within a narrow low‐frequency bandwidth, and they will be applied to development of automatic detection and classification algorithms as foundational data in future studies.

According to Thomas and DeMaster (1982), the diurnal pattern of underwater vocalizations is negatively correlated with the haul‐out pattern. In other words, the number of vocalizations was greater between 19:00 and 06:00 and lower between 09:00 and 18:00, as the species spends more times in the water at night. According to our results, the vocalization rate and sound pressure levels were relatively low from 05:00 to 17:00 on December 10, and were higher before and after, which was similar to the trend observed in the previous study but with a time shift. The persistence of elevated call rates and sound pressure levels from 05:00 to 17:00 on December 11 suggests that further validation of the diurnal pattern through long‐term observation is necessary. However, our measurements were conducted at higher latitudes and later in the year when day and night conditions are indistinct than those of Thomas and DeMaster (1982), which may explain the differences compared to the trend. The results from the Perennial Acoustic Observatory in the Antarctic Ocean (PALAOA) conducted from January 2006 to January 2007 showed that the leopard seal was most vociferous on 16 December (Van Opzeeland et al., 2010). Although acoustic data collected over 54 h in this study are insufficient for characterizing diurnal patterns and their correlations with environmental data, they were measured when vocalization rates might be near the peak so that leopard seal vocalizations could be the dominant sound source. The proportion of call types presented in Section 3.2 was subject to limited comparative analysis due to differences in call type classification from the previous study. The absolute call counts per minute were 3.7 (±0.03 SE). By call type, they were 1.2 (±0.02 SE) for HDT, 0.4 (±0.01 SE) for MST, 1.3 (±0.02 SE) for LDT, 0.5 (±0.01 SE) for HST, 0.3 (±0.01 SE) for DT, and 0.1 (±0.01 SE) for triple ascending trill. These values were significantly lower than those reported by Shabangu and Rogers (2021), because we extracted only calls that the structure could be clearly identified, which likely excluded calls with low signal‐to‐noise ratios. Furthermore, we were unable to collect environmental data such as sound speed profiles and water depths in the field, and long‐term PAM data are required to facilitate a more comprehensive analysis correlating these environmental variables with acoustic data.

## 5 CONCLUSION AND IMPLICATIONS

Underwater passive acoustic monitoring was conducted at Inexpressible Island in the Ross Sea region marine protected area for 3 days during the austral summer season, and we found that leopard seal vocalizations were the dominant sound sources in this region. Leopard seal underwater vocalizations, known to be relatively stereotyped, can be categorized into six call types based on previous studies, and their acoustic characteristics are presented. Among the low‐frequency vocalizations, we have termed a new call type, triple ascending trill, which consists of three trill parts in a frequency range of approximately 400 to 1540 Hz. These observations were supported by UAV and monitoring camera data collected together during the underwater acoustic measurements. Our research includes passive acoustic monitoring results that were conducted in a region, which has limited human access in the ASPA and no prior acoustic observations. We have applied state‐of‐the‐art techniques, which can be effective for pinnipeds, to research Antarctic marine mammals, as vocalizations can be recorded both underwater and out of the water, unlike those of whales. Our results can be applied to studies that estimate the population and distribution of marine mammals by measuring their acoustic habitat and could contribute to understanding the Antarctic marine ecosystem from a soundscape ecology perspective.

## AUTHOR CONTRIBUTIONS

**Dong‐Gyun Han:** Conceptualization (lead); formal analysis (lead); investigation (lead); visualization (lead); writing – original draft (lead); writing – review and editing (lead). **Jee Woong Choi:** Supervision (supporting); validation (supporting). **Jong‐U Kim:** Supervision (supporting); validation (supporting). **Jeong‐Hoon Kim:** Funding acquisition (equal); project administration (equal); supervision (equal); validation (equal). **Hyoung Sul La:** Conceptualization (equal); data curation (equal); supervision (equal); validation (equal); writing – review and editing (equal).

## FUNDING INFORMATION

This research was supported by a grant from the Korea Institute of Marine Science & Technology Promotion (KIMST), funded by the Ministry of Oceans and Fisheries (KIMST 20220547), and by a grant from the National Research Foundation (NRF), funded by the Korea government (MSIT) (2020R1A2C2007772).

## CONFLICT OF INTEREST STATEMENT

The authors declare that they have no competing interests.

## Supporting information


Figure S1.

## Data Availability

Acoustic data used in this study are available at the Korea Polar Data Center (https://kpdc.kopri.re.kr/search/cc13416e‐0129‐42b8‐9fc7‐d9237504eab3) and a reasonable request from the corresponding author.
