# Supplementary material for: Underwater soundscape in Seaview Bay, Antarctica, and triple ascending trill of the leopard seal (Hydrurga leptonyx) underwater vocalizations
Source: Ecol Evol. 2024 Jul 25;14(7):e70038. doi: 10.1002/ece3.70038 (PMC11272605; doi:10.1002/ece3.70038)
Supplement: Supplementary file 1 — Figure S1. [file ECE3-14-e70038-s001.docx]

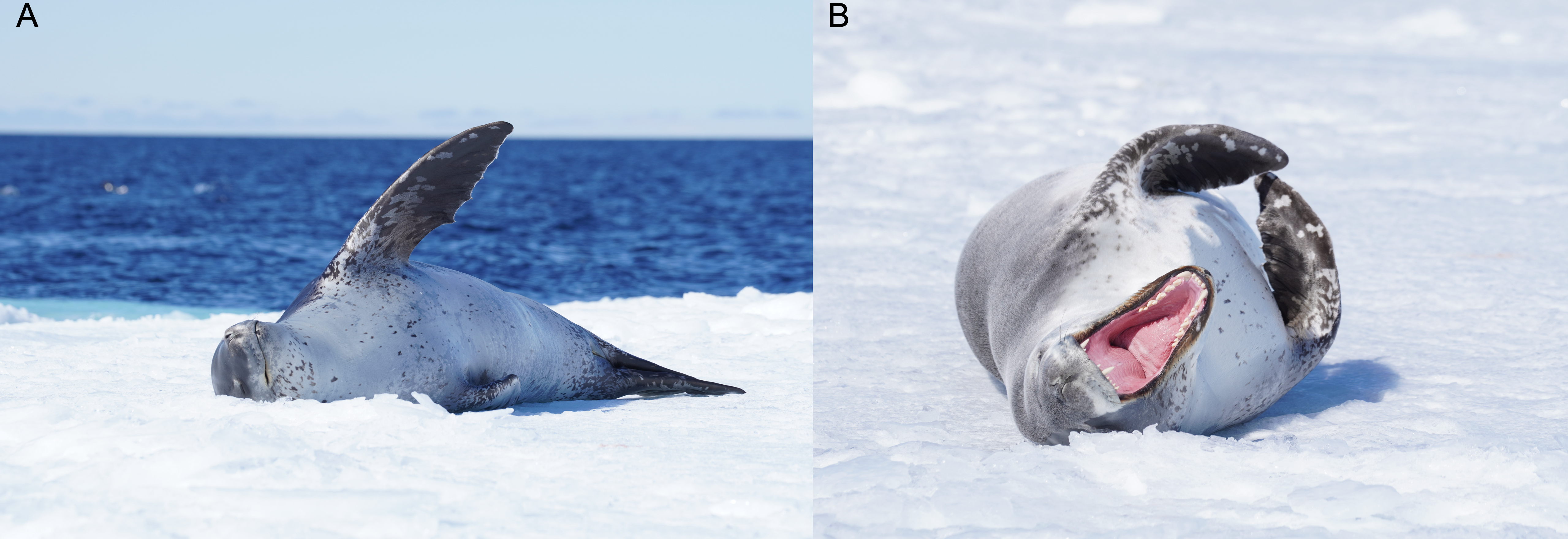


Figure S1. Leopard seal photos, which are the same entity in the expanded image of a red square box in Figure 1C on the pack ice. (A) Whole-body image of a leopard seal (female) discovered on the pack ice during acoustic measurements. (B) A close-up image showing the upper body.


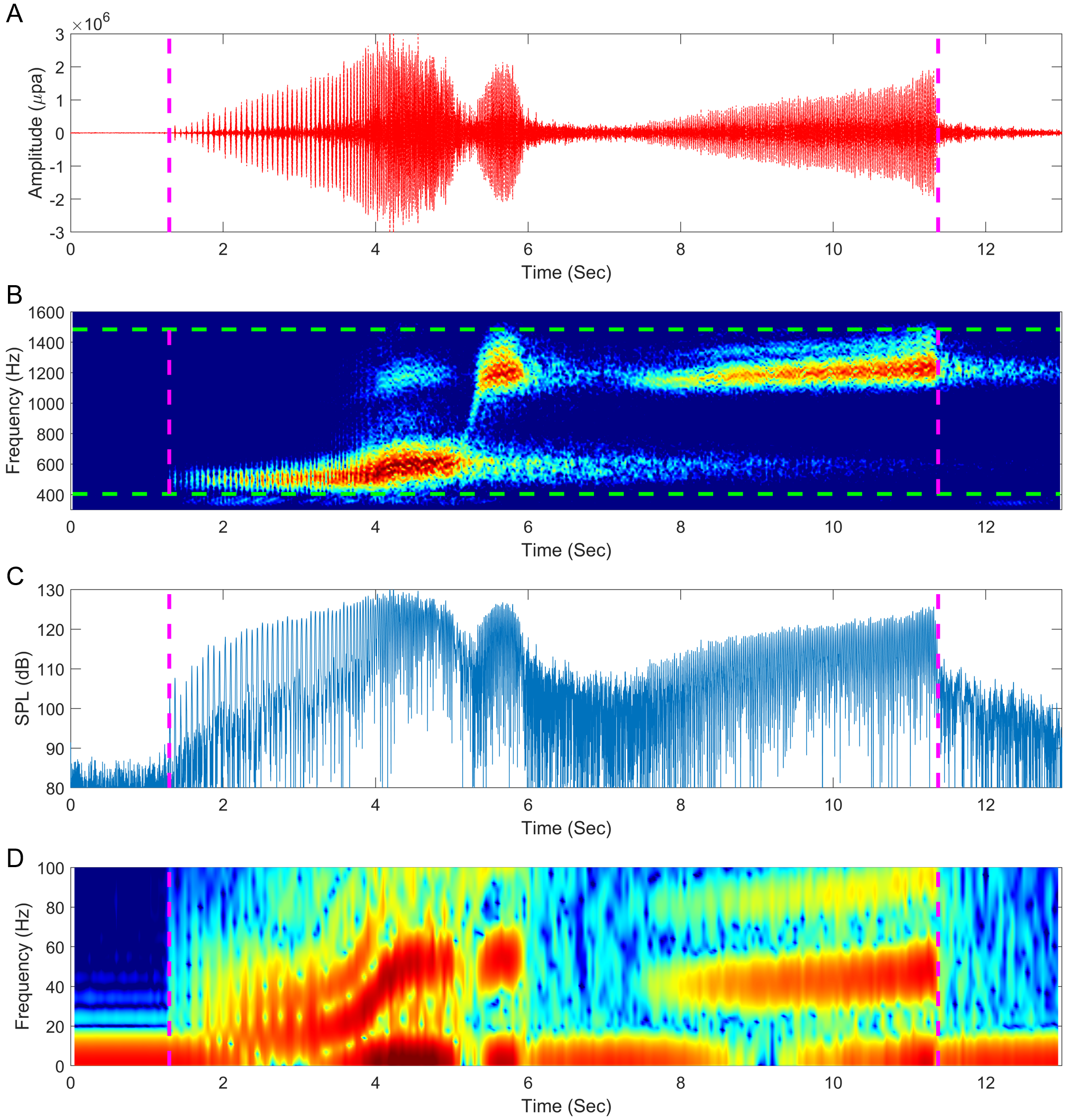


Figure S2. Analysis results of the ascending trill as a function of time. (A) Bandpass filtered waveform, (B) spectrogram (48,000 fast Fourier transform points and 4,800-point Hanning window), (C) sound pressure level, and (D) spectrogram of envelope waveform representing frequency modulation (96,000 fast Fourier transform points and 19,200-point Hanning window). Magenta dashed lines represent the beginning and ending points of call, and yellow green dashed lines represent maximum and minimum frequencies.
